# Trustworthiness judgments and Borderline Personality Disorder: an experimental study on the interplay of happiness and trustworthiness appraisals and the effects of wearing face masks during the Covid-19 pandemic in Germany

**DOI:** 10.1186/s40479-022-00193-x

**Published:** 2022-11-03

**Authors:** Miriam Biermann, Anna Schulze, Franziska Unterseher, Marie Hamm, Konstantina Atanasova, Dagmar Stahlberg, Stefanie Lis

**Affiliations:** 1 Department of Psychiatric and Psychosomatic Medicine, Central Institute of Mental Health Mannheim, Medical Faculty Mannheim, Heidelberg University, J5, 68159 Mannheim, Germany; 2 Department of Clinical Psychology, Central Institute of Mental Health Mannheim, Medical Faculty Mannheim, Heidelberg University, J5, 68159 Mannheim, Germany; 3 School of Social Sciences, University of Mannheim, A5, 6, 68159 Mannheim, Germany

**Keywords:** Trustworthiness, Emotion recognition, Face masks, Borderline personality, Corona pandemic, Social judgments

## Abstract

**Background:**

Judging positive emotional states or the trustworthiness of others is important for forming and maintaining social affiliations. Past studies have described alterations in these appraisal processes in Borderline Personality Disorder (BPD), which might have been exacerbated during the Covid-19 pandemic by the requirement to wear face masks. In the present study, we investigated in an online-survey a) whether social judgments are particularly strongly affected in individuals with BPD when they have to judge happiness and trustworthiness in facial stimuli covered by a mask, b) whether appraising a positive emotional state affects trustworthiness appraisals differentially in BPD and healthy individuals and c) whether social judgments are related to how individuals with BPD experience wearing masks during the pandemic.

**Methods:**

Participants (67 HC, 75 BPD) judged happiness and trustworthiness of faces with calm expression with and without masks. Additionally, data on participants’ confidence in their judgments, the experience of the burden induced by wearing masks, the protective benefits of masks, and compliance to wearing masks were collected.

**Results:**

Happiness and trustworthiness were evaluated less confidently and less intense in the BPD group compared to HC. Masks reduced happiness and trustworthiness ratings in both groups. Lower happiness appraisals contributed to lower trustworthiness appraisals except for those with BPD and low levels of symptom severity. Lower trustworthiness ratings were associated with a higher burden, attributing a lower benefit to masks and lower compliance with wearing masks in BPD.

**Conclusions:**

Masks do not exacerbate deficits in social judgments. However, lower trustworthiness appraisals in general were linked with more negative evaluations of wearing masks in the BPD group.

**Trial registration:**

The aims and hypotheses were preregistered together with the design and planned analyses (https://aspredicted.org/f5du7.pdf). For findings of an additionally preregistered research question on the impact of adverse childhood experiences see supplementary material.

**Supplementary Information:**

The online version contains supplementary material available at 10.1186/s40479-022-00193-x.

## Background

One of the core symptom domains in Borderline Personality Disorder (BPD) are pervasive interpersonal dysfunctions [1]. Studies on impairments of cognitive processes relevant for social functioning have revealed alterations in social judgments in BPD. Examples are changes in how individuals with BPD evaluate the quality and intensity of emotional states and attribute trustworthiness to facial stimuli (e.g., [2, 3]). Recent studies revealed similar impediments of social judgments through wearing mouth-nose covers (MNC) during the Covid-19 pandemic (e.g., [4–6]). Accordingly, it is obvious to hypothesise that pandemic-related requirements like wearing masks, particularly affect those individuals who already suffer from social-cognitive impairments in the context of mental disorders (e.g., [7]). The current study investigated the effects of masks on social judgments in BPD and their association with the evaluation of real-life experiences in wearing masks during the Covid-19 pandemic.

The face is an important source of information people use to recognise others’ emotional states, and infer complex social judgements, such as others’ trustworthiness. The appraisal of a facial emotion and attributed trustworthiness are closely related. For example, people ascribe a higher trustworthiness to happy faces [8, 9]. This implies that the ability to recognise positive emotional expressions is important for trusting others and accordingly, for subjective well-being and successful social interactions [10].

Individuals with a diagnosis of BPD experience positive social signals, such as another person’s smile, less intensively, feel less confident in their evaluation, and tend to misclassify faces with a subtle expression of happiness [11–15]. These impairments persist even after symptomatic remission [16, 17]. Moreover, BPD is related to issues with interpersonal trust (see for reviews: [3, 18]). Evidence is based on self-report questionnaires (e.g., [19]) and experiments, where individuals with BPD assessed facial stimuli as less trustworthy compared with healthy individuals (HC) (e.g., [20–22]). So far, there is a lack of studies that investigate, whether the link between appraising a positive emotion and trustworthiness exists in BPD to a similar extent as in HCs and whether impairments in the evaluation of a positive emotional expression of facial stimuli might thereby contribute to lower trustworthiness appraisals in BPD. A recent study by Fertuck & Grinband [20] focusing on negative emotional expressions revealed no association between fearfulness appraisals and untrustworthiness appraisals in BPD. This is in line with findings on trust behaviour, suggesting that emotional social cues are less sufficient to guide BPD patients’ trust behaviour compared with HCs [23, 24]. However, there is an ongoing debate whether trust and distrust constitute opposite poles of a continuum, or whether both rely on different functional systems [25]. In case of different functional systems, alterations in the interplay between trustworthiness judgments and emotional cues in mental disorders might differ depending on the valence of emotional social signals, i.e. on whether a positive social cue like a smile signals trustworthiness or a negative social cue such as clenching the jaws signals untrustworthiness. Therefore, further studies are needed to investigate the interplay between appraisals of positive emotional expressions and trustworthiness appraisals in BPD.

During the Covid-19 pandemic, behavioural restrictions such as wearing masks and social distancing affected everyone’s everyday social life. In particular, wearing a mask places special demands on social encounters: When facial features are partially occluded by masks, especially the recognition of emotional states such as joy is faultier [5, 26, 27]. These findings can be explained by previous studies on facial emotion processing [28, 29]: Especially for the recognition of joy, people allocate less time to assessing the eye region but more time to the lower part of a face that is covered by a mask. In a recent study, we could show that masks also resulted in a drop of trustworthiness appraisals for smiling faces [4, 6]. However, this effect of masks was less strong compared with judging happiness, most probably since the eye region of a face is more important than the mouth region in judgments of trustworthiness [30]. The finding that 47% of the variance of change in trustworthiness appraisals induced by masks was shared with changes in happiness ratings emphasises the influence of the appraisal of a positive emotional state on the appraisal of trustworthiness [4].

Being confronted with others wearing masks is not only an additional obstacle on social judgments, but also accentuates the uncertainty people experience during their judgments [5]. Several studies revealed that BPD patients are less confident when judging emotional cues compared with HCs and that this difference between groups might be accentuated with increasing difficulty of the judgement [14, 31, 32]. Thus, one might hypothesise that higher task demands induced by masks affect people with BPD more strongly regarding confidence during social judgements compared to HC.

Beyond the influence masks have on trustworthiness ratings by hampering the evaluation of emotional states, peoples’ attitudes towards masks also modulate the effects of masks on social judgments: Attributing protective effects to masks and experiencing a low burden during interactions with people wearing masks were related to smaller changes in appraisals of faces as being less happy and less trustworthy induced by masks [4]. So far, the association between attitudes towards masks and the effects of masks on social judgments has not been investigated in individuals with mental disorders.

In the present online study, we addressed two topics to contribute to the understanding of trustworthiness impairments in BPD in general, as well as during the Covid-19 pandemic. First, we were interested in the interplay between the appraisal of positive emotional states and trustworthiness and how social judgments such as the evaluation of happiness and trustworthiness are influenced by covering the presented facial stimuli with a face mask in individuals with BPD. We hypothesised that (a) masks influence social judgments to a higher extent in a BPD compared to a HC group. Due to the higher relevance of the mouth region for happiness judgments, we expected that masks result in a stronger decrease in intensity ratings for happiness than trustworthiness ratings. Moreover, we expect that (b) the intensity of a positive emotional state ascribed to a facial stimulus predicts the judgment of trustworthiness in the BPD group to a lower extent than in HC. In regard to the confidence participants experience during social judgments, we expected that (c) masks will reduce confidence in the BPD group to an even higher extent than in the HC group.

Second, we were interested in how individuals with BPD experience the burden induced by mask wearing during social encounters in everyday life and whether this burden is related to their social judgments. We expected that (d) individuals of the BPD group experience a higher burden during real-life social contacts due to masks compared with HCs and that a higher burden is related to stronger effects of masks on social judgments and the individuals’ confidence in these judgments. Additionally, we explored the relation between social judgment and attitudes towards masks in regard to their perceived efficiency in protecting from infection, as well as compliance to wearing masks.

Due to lockdown regulations at the time of the study, we recruited individuals of the BPD or HC groups of former research projects. At the time of testing, participants presented with a range of current symptom and function levels.

## Methods

The study was conducted between February 13, and April 4, 2021, when Germany was in lockdown under strict measures, which included closing most public facilities, wearing FFP2 or surgical masks in all public spaces and limiting social contact to one person from another household. Due to the lockdown the study was done as an online survey via unipark. Participating in the survey was possible through computers and smartphones.

### Participants

Participants were recruited from the database of the central project of the KFO 256, a Clinical Research Unit funded by the German Research Foundation dedicated to investigating mechanisms of disturbed emotion processing in BPD [33]. We included 149 women in this online study of which 67 were healthy controls and 75 individuals who had met the *Diagnostics and Statistical Manual of Mental Disorders* (DSM-IV) [34] diagnosis of BPD in the past, that is, met at least five of the nine DSM–IV criteria for BPD, assessed by trained clinical psychologists using the International Personality Disorder Examination (IPDE) [35]. For further details on the recruitment procedure, see supplementary material A. All individuals provided written informed consent before participating in the survey. The study was approved by the Research Ethics Board of the Medical Faculty Mannheim of Heidelberg University. The participants received a small fee for participating.

We characterised the samples by assessing sociodemographic features, psychopathology, and general trust propensity. We measured BPD symptom severity with the short version of the Borderline Symptom List (BSL-23) [36], the level of BPD features with the German version (VEI-BOR) [37] of the Borderline Scale from the Personality Assessment Inventory (PAI-BOR) [38] and severity of depressive symptoms with the German version [39] of the Beck Depression Inventory-II (BDI-II) [40].

The severity of childhood trauma was based on self-reports measured with the German version [41] of the short form of the Childhood Trauma Questionnaire (CTQ-SF) [42]. Interpersonal trust propensity was assessed with the *Kurzskala Interpersonelles Vertrauen* (KUSIV-3) [43]. For further details, see supplementary material B.

### Experimental task and stimulus material

During the experimental task, each participant assessed how intensely a facial stimulus expressed happiness and trustworthiness (within-subject factor: task) and how confident they were in the judgment. Additionally, we manipulated the visible part of the faces by presenting each face with and without a FFP2 mask (within-subject factor: mask). Participants indicated their responses on a 7-point Likert scale ranging from 1 to 7 (‘How strongly expresses the face happiness/trustworthiness? ’ *not at all* to *very much*).

For facial stimuli, we used calm facial expressions with a straight gaze from 12 different stimulus characters (50% men, 50% women) from the Interdisciplinary Affective Science Laboratory Face Set (IASLab Face Set; IDs of the selected face stimuli: F02, F06, F11, F22, F30, F31, M01, M05, M07, M09, M10, M19). Calm faces have the same valence as happy faces, but a lower level of arousal (see affective circumplex model [44]). Calm faces are rated as happier and more trustworthy compared with neutral faces [8]. In contrast to happy faces, calm faces display no prototypical emotional expressions, which are often associated with ceiling effects and which have been criticised in the past as being of low ecological validity as neutral faces [8]. In contrast to happy faces, calm faces display no prototypical emotional expressions, which have been criticised in the past as being of low ecological validity [45]. Each image was edited with GIMP photo editing software to apply a FFP2, resulting in 24 different stimuli. All faces were presented as greyscale images. For exemplary stimuli, see supplementary material Fig. S1.

Participants rated happiness and trustworthiness together with their confidence in two separate blocks. Each block was split into two sub-blocks, presenting each of the 12 stimulus characters of which six were presented with masks and six without masks. The trials within each sub-block, the order of the sub-blocks within each block, and the types of social judgement were counterbalanced across participants. Please note that while this prevents a differential impact of time-related confounds on task performance, it might also influence the rating in case of asymmetrical transfer effects. The experimental design was adapted from the one used in [4].

### Cognitions, emotions, and safety behaviour related to the pandemic

We asked participants to answer a set of questions related to the experience of wearing masks during the pandemic (adapted from [4]). Questions referred to the physical burden as well as the emotional burden and relief induced by masks during social encounters, the protective benefits ascribed to wearing masks, as well as compliance with wearing masks. All items were answered on a 6-point Likert scale (range 1 *not at all* to 6 *very much*). For more details, see supplementary material D.

For further characterisation of the samples, we additionally asked particpants of whether.

### Statistical analyses

Social judgements and confidence ratings were analysed in separate 2 × 2 × 2 mixed-ANOVA designs with the repeated-measurement factors mask (without/with FFP2) and task (happiness/trustworthiness) and the between-subject factor group (BPD/HC). Assumptions for ANOVA were tested by tests of normality and inspection of QQ plots. We explored the nature of interaction effects by 2 × 2 sub-designs.

To estimate the extent to which the experienced intensity of happiness predicts trustworthiness ratings, we calculated a linear regression analysis with trustworthiness ratings as the dependent variable and happiness ratings and group membership (dummy coded with HCs = 0 and BPD = 1) as predictors.

To analyse whether the HC and BPD groups differed in the extent to which they experienced masks as a burden during real-life social encounters and whether this experience co-varies with the changes in social judgments related to masks (difference between ratings with and without a mask), we compared both groups using Mann-Whitney *U* tests, calculated Spearman correlation coefficients (*r*_*s*_) separately for each group, and compared these coefficients to test for differences in these relationships. In additional exploratory analyses, we conducted corresponding analyses for the protective benefit people ascribed to wearing masks and their compliance to wearing masks. Analyses were performed with SPSS 27 or MATLAB R2019a.

### Preregistration

The aims and hypotheses were preregistered together with the design and planned analyses (https://aspredicted.org/f5du7.pdf). For findings on an additionally preregistered research question on the impact of adverse childhood experiences see supplementary material.

## Results

### Sample description

The groups were balanced for age and education (all *ps* > .1). The BPD group reported a higher level of BPD symptoms (BSL-23), BPD features (VEI-BOR) and depressive symptoms (BDI-II), as well as a lower interpersonal trust propensity, compared to the HC group (all *ps* < .001). We analysed the frequency of severity categories of BPD symptoms in both samples following the suggestions by [46] for BSL-23 symptom severity categories and [38] for VEI-BOR categorisation of individuals with and without a clinically relevant level of BPD features. These analyses revealed a broad range of BPD symptom severity in the BPD group with 19 individuals reporting BSL-23 scores in the range of low (*N* = 3) or mild (*N* = 16) symptom severity and 15 participants with a level of BPD features below the cut-off for clinical significance. In the BPD group, a higher percentage of participants (68.00%) reported to be currently in psychotherapeutic or psychopharmacological treatment compared to the HC group (1.50%, χ^2^ = 67.4, *p* < .001). 47% of the BPD individuals with low to mild symptoms were currently in treatment. According to Corona-related measures, most of the participants had not suffered a confirmed Covid-19 infection themselves (HC: 85.1%, BPD: 73.3%, Χ^2^ = 2.93, *p* = .102) but almost every second person had a close other who had been infected (HC: 53.7%, BPD: 57,3%, Χ^2^ = 0.19, *p* = .736). They reported that they wear masks comparably often as other people around them (HC: *M* = 3.6, *SD* = 1.13, BPD: *M* = 3.5, *SD* = 1.20, MW-U = 2496.5, *p* = .946, evaluated on a 7-point Likert scale ranging from 1 ‘more often’ to 7 ‘less often’). For further details, see Table 1 and Table S1.

**Table 1:** Sample description

	HC *N* = 67		BPD *N* = 75		Test statistics	*P*
	*M*	*SD*	*M*	*SD*		
Age^a^	33.06	7.13	33.72	8.53	−0.50	.620
Education (%)^c^					2245	.119
- Vocational school	11.90	–	20.00	–		
- Comprehensive/Academic- secondary school	82.10	–	77.30	–		
- Other	6.00	–	2.70	–		
Psychopathology						
BSL-23^a^	0.28	0.29	1.71	0.91	−12.82	< .001***
VEI-BOR^a^	17.00	8.07	46.25	10.88	−18.02	< .001***
BDI-II^a^	7.69	5.94	25.53	14.00	−10.07	< .001***
Childhood traumatization						
CTQ^a^	28.69	3.29	58.97	19.80	−13.05	< .001***
Interpersonal trust propensity						
KUSIV-3^a^	3.81	0.56	2.89	0.89	7.49	< .001***
Current affective state						
SAM-valence^b^	3.01	1.42	4.89	1.86	1034	< .001***
SAM-arousal^b^	6.39	1.83	4.97	1.86	1470	< .001***
Evaluations of wearing masks						
Somatic burden^b^	2.34	1.52	3.07	1.56	1798	.003**
Emotional burden^b^	3.60	1.42	3.30	1.47	2275	.328
Relief^b^	2.80	1.46	3.21	1.48	2110	.097(*)
Protective benefit^b^	4.52	1.10	4.08	1.09	1983	.028*
Compliance^b^	5.07	0.83	4.95	0.95	2420	.698

^a^Student’s *t*

^b^Mann-Whitney *U*

^c^Chi-Square

### Appraisal of happiness and trustworthiness

**Fig. 1:**
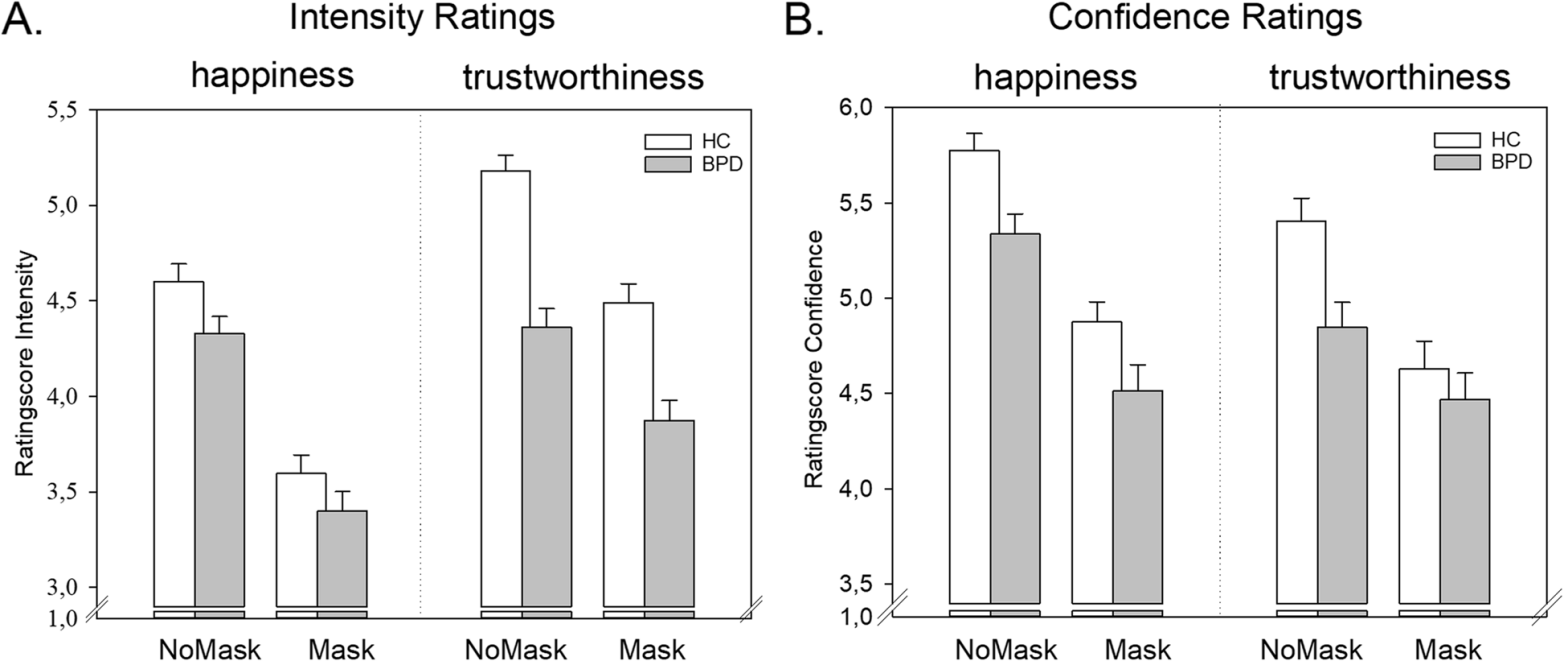
Happiness and trustworthiness ratings for faces with and without masks in the HC and BPD group and the participants’ confidence in their judgments

Individuals with BPD rated faces as less happy and less trustworthy than HCs (group: *F*(1, 140) = 22.38, *p* < .001, η_p_^2^ = .14; Fig. 1). The difference between groups was larger for trustworthiness than for happiness ratings (task × group: *F*(1, 140) = 13.00, *p* = .001, η_p_^2^ = .09; happiness *t*(140) = 1.98, *d* = 0.33; trustworthiness *t*(140) = 5.82, *d* = 0.98). HC individuals rated trustworthiness higher than happiness, compared with the BPD group (comparison of tasks; HC: *t*(66) = − 8.80, *d* = − 1.11; BPD: *t*(74) = − 2.48, *d* = − 0.32). Covering a face with a mask led to lower happiness and – to a smaller extent – lower trustworthiness in both groups (mask: *F*(1, 140) = 201.30, *p* < .001, η_p_^2^ = .59; task × mask: *F*(1, 140) = 44.71, *p* = .001, η_p_^2^ = .24; task × mask × group: *F*(1, 140) = 1.21, *p* = .274, η_p_^2^ = .01; group × mask: *F*(1, 140) = 1.67, *p* = .198, η_p_^2^ = .01). See summary of results, supplementary material, Table S2.

Association with BPD symptom severity: To explore the effects of BPD symptom severity on social judgments, we repeated the analysis for the BPD group with the BSL-23 score as covariate. Analyses revealed that ratings of intensity were lower for individuals with higher BSL-23 scores (BSL: *F*(1,73) = 11.91, *p* < .001). At trend level, higher BSL-23 scores predicted a stronger drop in intensity ratings for faces covered by a mask (Mask x BSL: *F*(1,73) = 3.43, *p* = .068). For further details, supplementary material G, Table S3, Fig. S2.

### Happiness appraisals as predictor of trustworthiness

Linear regression analysis revealed that higher happiness ratings predicted higher trustworthiness ratings without evidence for a differential effect in both groups (Table 2, model 1, *R*^*2*^ = .32, *R*^*2*^_*adj*_ = .30, *F*(3,138) = 21.21, *p* < .001).

**Table 2:** Results of linear regression analyses

	*b*	*SE*	β	*t*	*P*	*95% CI*
**Model 1**						
Intercept	4.78	0.09		56.49	< .001***	[4.61, 4.95]
Group	−0.62	0.12	−.38	− 5.35	< .001***	[−0.85, − 0.39]
Happiness	0.32	0.12	.39	3.61	< .001***	[0.14, 0.49]
Group × happiness	−0.05	0.12	−.05	−0.45	.653	[−0.28, 0.18]
**Model 2 BPD group**						
Intercept	4.19	0.08		52.43	< .001***	[4.03, 4.35]
Happiness	0.07	0.09	.08	0.76	.452	[−0.11, 0.24]
BSL	− 0.20	0.08	−.25	−2.53	.014*	[−0.37, − 0.04]
Happiness × BSL	0.30	0.08	.42	3.87	< .001***	[0.14, 0.45]

*Happiness*: ratings of intensity of happiness (*z*-transformed), *BSL* BSL-23 scores (*z*-transformed)

Association with BPD symptom severity: We explored the effects of BPD symptom severity as modulating factors in the BPD group by adding BSL-23 scores and its interaction with happiness judgments as predictors for trustworthiness judgments. The regression model (Table 2, model 2, *R*^*2*^ = .33, *R*^*2*^_*adj*_ = .30, *F*(3,71) = 11.65, *p* < .001) revealed an effect of symptom severity in addition to an interaction between BSL-23 scores and happiness ratings: higher happiness ratings predicted higher trustworthiness ratings depending on higher symptom severity.

### Confidence in social judgements

The confidence in social judgments decreased when faces were covered by a mask compared with faces without a mask (mask: *F*(1, 140) = 155.90, *p* < .001, η_p_^2^ = .53). While there was no difference in the decrease of confidence induced by masks between groups for happiness ratings, the decrease was stronger in the HC group compared with the BPD group for trustworthiness ratings (group × task × mask: *F*(1, 140) = 4.63, *p* = .033, η_p_^2^ *=* .03; analysis of 2 × 2 ANOVA sub-designs: trustworthiness ratings group × mask: *F*(1, 140) = 9.00, *p* = .003, η_p_^2^ = .06, group: *F*(1,140) = 4.03, *p* = .047, η_p_^2^ = .03; happiness ratings group × mask: *F*(1, 140) = 0.28, *p* = .600, η_p_^2^ = .002, group: *F*(1,140) = 7.70, *p* = .006, η_p_^2^ = .05, mask: *F*(1,140) = 146.49, *p* < .001, η_p_^2^ < .01). While confidence in judging trustworthiness in faces without masks was lower in the BPD group compared with the HC group (*t*(140) = 3.09, *p* = .002, *d* = 0.52), there was no difference for faces with masks (*t*(140) = 0.80, *p* = .424, *d* = 0.14). For summary of results, see Table S2.

Association with BPD symptom severity: Exploring the effects of BPD symptom severity in the BPD groups revealed no statistically significant differences between the low and high symptom severity groups (all *ps* > .195, Table S3).

### Evaluations of wearing masks

The BPD group reported a higher physical burden induced by masks compared to HCs. While both groups did not differ in the emotional burden experienced, the BPD group reported a stronger emotional relief due to masks at trend level (Table 1).

An exploratory analysis revealed that the protective function ascribed to masks was lower in the BPD group compared to the HC group. Nevertheless, both groups did not differ in the compliance of wearing masks (Table 1).

### Correlation between the evaluation of wearing face masks and changes in social judgments induced by masks

There was no correlation between changes in happiness and trustworthiness appraisals or the confidence in these judgments induced by masks and the burden, the relief, the attribution of a protective function or the compliance associated with wearing masks (all *ps* > .100; Table S4).

However, additional exploratory analyses of the overall happiness and trustworthiness appraisals revealed that lower trustworthiness appraisals were associated with a higher somatic burden and lower relief through wearing masks, as well as ascribing lower protective functions to masks and being less compliant with wearing masks in the BPD group, but not in the HC group (Table 3). Exploratory analyses of partial correlation coefficients including BSL-23 scores as covariate revealed that these relationships within the BPD group cannot be explained by variations in BPD symptom severity (Table S5). There were no significant associations with the appraisal of happiness or the confidence in social judgments (for details, Tables S6 and S7).

**Table 3:** Trustworthiness ratings and the evaluation of different facets of wearing masks

	HC		BPD		Comparions of *r*_*s*_ between groups	
	*r*_*s*_	*p*	*r*_*s*_	*p*	*z*	*p*
Somatic burden	−.13	.305	−.40	< .001***	1.69	.044*
Emotional burden	−.00	.982	.19	.110	−1.41	.128
Relief	−.01	.951	.26	.026*	−1.59	.054(*)
Protective benefit	−.03	.839	.29	.010*	−1.92	.028*
Compliance	.04	.735	.32	.005**	−1.70	.045*

Spearman correlations (*r*_*s*_) and comparison of correlation coefficients between groups are reported

## Discussion

In the present study, we investigated social judgments of facial stimuli with positive emotional expressions and the effects of covering these faces with a mask. Our findings revealed that the BPD group experienced faces with and without masks as less happy and, to an even stronger extent, as less trustworthy compared with HCs. In contrast to our hypotheses, covering the face with a mask resulted in a drop of comparable extent in the intensity of happiness and trustworthiness in both groups. While confidence in their judgments was lower in the BPD group than in HC for unmasked faces, healthy individuals felt comparably uncertain about their judgments of trustworthiness in masked faces as individuals of the BPD group. Positive emotional expression of a face predicted trustworthiness ratings to a similar extent in both groups. However, in the BPD group, this relationship was attenuated with decreasing symptom severity. Changes in social judgments induced by masks were not related to the evaluations of different aspects of wearing masks during the pandemic. However, in the BPD group, lower trustworthiness appraisals were associated with a higher somatic burden, lower emotional relief when wearing masks, attributing lower protective functions to masks, and being less compliant with wearing masks. This supports the relevance of dysfunction in interpersonal trust for cognition, emotions and behaviour during the Covid-19 pandemic.

Impairments in judging cues relevant for building and maintaining social affiliations in people with BPD have been shown in several studies (e.g., [11–15, 17, 20–22]). In line with these findings, we found lower ratings of a positive emotional state in calm faces and – even more pronounced – reduced trustworthiness appraisals of these faces in BPD, compared to HCs. However, in contrast to our hypotheses, there were no differences in the decrease of happiness and trustworthiness appraisals induced by hiding the lower part of the face behind a mask between the groups. While this suggests that wearing masks during the pandemic do not aggravate changes in social judgments in BPD, it raises some doubt on whether changes in the attribution of a positive valence to social cues are indeed determined by deficits in decoding facial features in BPD. It seems important to mention that faces wearing a mask are perceptually very similar to the stimuli used in the Reading the Mind in the Eyes Task (RMET; [47]). The RMET is a well-established task to measure mental state decoding and has been used in several studies comparing individuals with BPD and HC. A recent meta-analysis revealed no differences in RMET performance between individuals with BPD and healthy controls in general [48]. However, a review by Richman and Unoka [49] identified moderating effects of co-morbidities and the valence of the RMET trials: individuals with a BPD performed worse particularly in positive valence tasks of the RMET compared with individuals with a comorbid major depression. While we did not analyse the effects of co-morbidities, our findings point to the severity of BPD psychopathology as an additional moderating factor.

The appraisal of a positive emotional state predicted around 30% of the variance in the appraisal of the more complex social judgment trustworthiness. This confirms the interplay between both types of social judgments described in previous studies [8, 9, 50]. This finding implies that experiencing a positive emotional social cue as less intense contributes at least partially to the experience of lower trustworthiness in BPD. Most importantly, this is the case for those individuals in the BPD group with a high symptom severity. Together with a recent study by [20] that did not find a relationship between appraisals of fear and trustworthiness, our findings underline the importance of taking into account the distinction between trust and distrust as different functional systems [25] when investigating trust issues in BPD. Whether strengthening the appreciation of positive social cues in others might provide a promising avenue to indirectly target trust issues in those people with BPD who have a generally negative view of others’ trustworthiness has to be investigated in future studies. However, our findings also suggest that the interplay between different social-cognitive processes changes with symptomatic remission: The relationship between judging an emotional state and trustworthiness seen in healthy individuals was attenuated in those BPD individuals with low levels of BPD symptoms. This points to a change in the mechanism underlying altered social judgments over the course of the disorder and emphasises the need for further studies in BPD after symptomatic remission, to understand what might predispose individuals towards a re-occurrence of BPD symptoms.

Covering the faces with a mask resulted in a lower confidence during social judgments. However, in contrast to our hypotheses, differences in confidence ratings between groups varied depending on the social judgment. For judging the emotional state, confidence dropped in both groups to a similar extent. For trustworthiness judgments, only the HC group became less confident in the presence of masks, resulting in a comparably low confidence for both groups. Thus, social judgments were related to a higher uncertainty in BPD as shown in previous studies [14, 31, 32], but this uncertainty was not affected more strongly by wearing masks during social encounters than in HCs.

The importance of first impression trustworthiness appraisals in social life has been shown in many studies [51]. Our data suggest that they might also be relevant for cognitions, emotions and behaviour during the Covid-19 pandemic. Particularly in the BPD group, lower trustworthiness appraisals were associated with a higher somatic burden, a lower feeling of relief induced by masks, ascribing lower protective functions to masks, and being less compliant with wearing masks. Uncovering these relationships for the general trustworthiness individuals ascribe to a facial stimulus underlines the importance of issues with trust in BPD for every-day life.

Some limitations of the present study have to be addressed. These include the restricted generalisability of our findings, since only women were included in the current study. Moreover, we included individuals in the BPD group based on the diagnosis of a BPD in the past, independently of whether they currently met the DSM-5 criteria. BSL-23 scores suggested that 4% of the participants reported only low levels of BPD symptoms, implying that our sample also includes individuals currently in symptomatic remission. BPD is a personality disorder characterised by frequent changes between recovery and the reoccurrence of symptoms [52]. However even during recovery, the level of social functioning is reduced [16, 17, 53]. Our findings show that lower levels of BPD symptoms are linked to an overall more positive view of others. They also suggest that those suffering from particularly high levels of BPD psychopathology might even be more vulnerable to wearing masks. Since this finding was statistically only marginally significant, it has to be interpreted with care. However, our findings also reveal that the structure of mental processes changes with symptomatic remission that differs from healthy individuals. We wish to emphasise that our findings on the effects of masks might underestimate their influence in individuals with very high levels of BPD psychopathology. However, our findings might also have a higher generalisability to individuals with BPD independently of their current psychopathological state. Nevertheless, participating in research projects is always voluntary and the fact that only a subsample of the contacted individuals was willing to give their written consent, emphasises that findings of studies like the current one have to be interpreted with care due to a sampling bias. Moreover, further studies with clinical control groups are needed to investigate whether our findings are specific for BPD or capture transdiagnostic features shared in different mental disorders. Additionally, further studies are needed that address whether the observed changes can be generalised to faces displaying more intense emotions than the calm faces used in the current study. Beyond this, we focussed on explicit social judgments. Our data suggest that these judgements are related to behaviours during the pandemic. Nevertheless, further studies are required that investigate whether the effects of masks on implicit social judgments and behaviours differ between individuals with BPD and HC (see for studies in HC e.g., [54–56]). Finally, the study was implemented as an online-survey due to the restrictions imposed by the Covid-19 pandemic.

## Conclusions

Our data confirm impairments in appraisals of a positive emotional state and trustworthiness in BPD and point to changes in the interplay between these two types of social judgments that might be of particular importance when the acute symptoms of BPD are less intense. However, our data do not confirm that wearing masks during the Covid-19 pandemic affects individuals with BPD to a higher extent than healthy individuals, although there are some hints that this might be the case in individuals with very or extremely high levels of BPD symptoms. Finally, our data reveal the importance of alterations in trustworthiness appraisals, measured by an experimental task, for the evaluation of different facets of wearing masks during the pandemic and maybe most importantly, with the compliance of wearing masks in BPD.

## Supplementary Information

**Additional file 1.** A. Description of Recruitment Procedure. B. Description of the Self-report Questionnaires. C. Stimulus material. D. List of the mask-related Questions. E. Distribution of Levels of BPD Symptom Severity and BPD Features. F. Summary Statistics of 2 × 2 × 2 ANOVA. G. Summary Statistics of 2 × 2 ANOCVA in the BPD sample with BSL-23 covariate. H. Relationships between Social Judgments and Different Facets of Wearing masks. I. Effect of Childhood Abuse and Neglect on Social Judgments and Confidence During Judgements in BPD.

## Data Availability

The datasets used and/or analysed during the current study are available from the corresponding author on reasonable request.

## Abbreviations

BDI-II: Beck’s Depression Inventory II
BPD: Borderline Personality Disorder
BSL-23: Short version of the Borderline Symptom List
CTQ-SF: Short form of the Childhood Trauma Questionnaire
DSM: Diagnostic and Statistical Manual of Mental Disorders
HC: Healthy individual
IASLab Face Set: Interdisciplinary Affective Science Laboratory Face Set
IPDE: International Personality Disorder Examination
KUSIV-3: Kurzskala Interpersonelles Vertrauen
PAI-BOR: Borderline Scale from the Personality Assessment Inventory
SAM: Self-Assessment Manikin
VEI-BOR: German version of the Borderline Scale from the Personality Assessment Inventory
