# Supplementary material for: Trustworthiness judgments and Borderline Personality Disorder: an experimental study on the interplay of happiness and trustworthiness appraisals and the effects of wearing face masks during the Covid-19 pandemic in Germany
Source: Borderline Personal Disord Emot Dysregul. 2022 Nov 3;9:27. doi: 10.1186/s40479-022-00193-x (PMC9629878; doi:10.1186/s40479-022-00193-x)
Supplement: Supplementary file 1 — Additional file 1. A. Description of Recruitment Procedure. B. Description of the Self-report Questionnaires. C. Stimulus material. D. List of the mask-related Questions. E. Distribution of Levels of BPD Symptom Severity and BPD Features. F. Summary Statistics of 2 × 2 × 2 ANOVA. G. Summary Statistics of 2 × 2 ANOCVA in the BPD sample with BSL-23 covariate. H. Relationships between Social Judgments and Different Facets of Wearing masks. I. Effect of Childhood Abuse and Neglect on Social Judgments and Confidence During Judgements in BPD. [file 40479_2022_193_MOESM1_ESM.docx]

**Supplementary Material**

**Trustworthiness judgments and Borderline Personality Disorder: an**

**experimental study on the interplay of happiness and trustworthiness appraisals and the effects of wearing face masks during the Covid-19 pandemic in Germany**

Biermann, Miriam^a^; Schulze, Anna^b^; Unterseher, Franziska^b^; Hamm, Marie^b^; Atanasova,

Konstantina^b^; Stahlberg, Dagmar^c^; & Lis, Stefanie^a, b^

^a^Department of Psychiatric and Psychosomatic Medicine, Central Institute of Mental Health Mannheim, J5, D-68159 Mannheim, Germany / Medical Faculty Mannheim, Heidelberg University, J5, 68159 Mannheim, Germany.

^b^Department of Clinical Psychology, Central Institute of Mental Health Mannheim, J5, D-68159 Mannheim, Germany / Medical Faculty Mannheim, Heidelberg University, J5, 68159 Mannheim, Germany.

^c^Chair of Social Psychology, School of Social Sciences, University of Mannheim, A5,6, D-68159 Mannheim, Germany

# A. Description of Recruitment Procedure

From the database, we contacted 145 healthy women (HC) and 132 women who had met a *DSM-4* diagnosis of Borderline Personality Disorder (BPD). Criteria for the BPD group were that individuals were female and adult, had given their written consent to be contacted for further study participation, that the contact information was still valid and who had met the DSM IV diagnosis of BPD based on interviews conducted by trained clinicians. To achieve a sufficiently large sample size, we contacted all individuals who received the diagnosis within the last six years. Based on this selection, we draw a sample of healthy participants meeting the same criteria stratified for age and education.

Of the contacted individuals, 53 individuals (32 HC, 21 BPD) did not respond and 75 individuals (43 HC, 32 BPD) were not interested in participation or did not provide their written informed consent. Of the 149 participants (70 HC, 79 BPD), we excluded 7 participants (3 HCs and 4 BPD) from analyzes because they did not confirm to have completed the survey alone, to have answered honestly, had not participated in the experimental task, or reported to have not followed the instruction of the experimental task properly. Thus, the final dataset consisted of 142 participants (67 HC, 75

BPD). Please note that we contacted only HCs without a history of childhood traumatization (CTQ score below cut-offs for a female community sample [1], please see below).

# B. Description of the Self-report Questionnaires

We measured the BPD symptom severity with the short version of the Borderline Symptom List (BSL-23) [2]. In the current study, the BSL-23 (range 0 – 4 ) had a Cronbach’s α of α = .97 for the total sample, α = .95 in the BPD group, and α = .87 in HC group. The level of BPD features was measured using the German version (VEI-BOR) [3] of the Borderline Scale from the Personality Assessment Inventory (PAI-BOR) [4]. The VEI-BOR (range 0 – 72) in the current study had a Cronbach’s α of α = .95 in the total sample, α = .85 in the BPD group, and α = .78 in the HC group. Severity of depressive symptoms was measured using the German version [5] of the Beck Depression Inventory-II (BDI-II) [6]. In the current study, the BDI-II (range 0 – 63) had a Cronbach’s α of α = .96 in the total sample, α = .94 in the BPD group and α = .87 in HC group.

The severity of childhood traumatization was based on self-reports measured with the German version [7] of the short form of the Childhood Trauma Questionnaire (CTQ-SF) [8]. The CTQ is a self-report questionnaire, providing a composite score for trauma load (range 25 – 125) in the domains of emotional neglect, physical neglect emotional abuse, physical abuse, and sexual abuse (Cronbach’s α = .87). Please note that the CTQ was not measured in the current survey, but during previous study participation in the KFO-256.

Interpersonal trust propensity was assessed with the *Kurzskala Interpersonelles Vertrauen* (KUSIV-3) [9]. The KUSIV-3 is a three-item self-report questionnaire providing a total score for general interpersonal trust (e.g., ‘I am convinced that most people have good intentions.’, 5-point Likert scale, range of the total score 1 – 5).

# Stimulus material

#
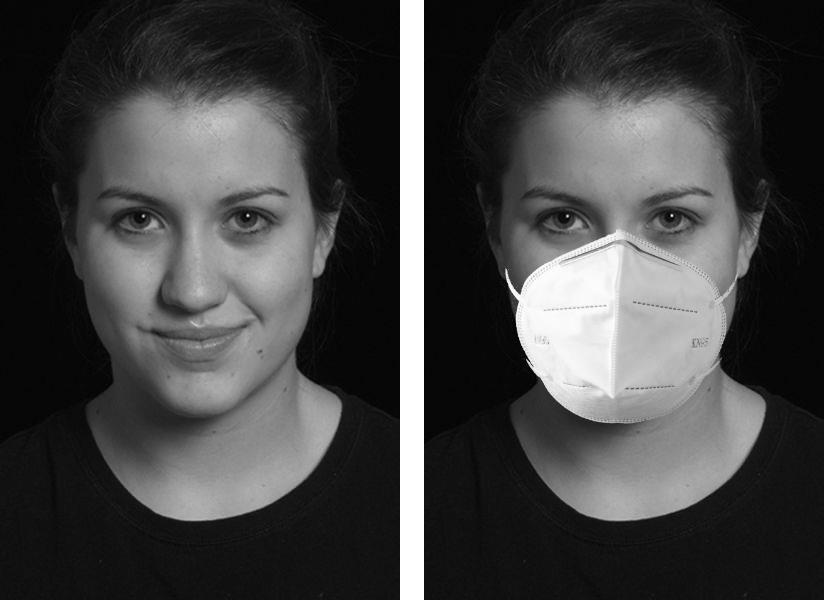
Fig. S1 Example of a facial stimulus without and with MNC^^[[1]](#footnote-1)^^

# D. List of the MNC-related Questions

*Burden*

‘How strong is the physical burden of wearing a mask?’

‘How much do social encounters become emotionally more difficult when you wear a mask?’ ‘How much do social encounters become emotionally more difficult when the others wear a mask?’

‘How much do social encounters become emotionally easier when you wear a mask?’ ‘How much do social encounters become emotionally easier when the others wear a mask?’

*Protective benefit through wearing MNC*

‘How strongly can you protect yourself by wearing a face mask?’

‘How strongly can you protect others by wearing a face mask?’

*Compliance with wearing MNC*

‘How often do you wear a face mask when it is mandatory?’

‘How often do you wear a face mask when doing so by your own free will?’

# Distribution of Levels of BPD Symptom Severity and BPD Features

# Table S1a Distribution of severity of BPD symptoms measured with the BSL-23

| Severity category | Range of BSL-23scores |  | | | In treatment | |
| --- | --- | --- | --- | --- | --- | --- |
|  |  | HC | | BPD | HC | BPD |
| None or low | 0.00 < 0.28 | 40 | | 3 | 0 | 1 |
| Mild | 0.28 < 1.07 | 25 | | 16 | 1 | 8 |
| Moderate | 1.07 < 1.87 | | 2 | 23 | 0 | 17 |
| High | 1.87 < 2.67 | | 0 | 20 | 0 | 15 |
| Very high | 2.67 < 3.47 | | 0 | 11 | 0 | 9 |
| Extremely high | 3.47 – 4.00 | | 0 | 2 | 0 | 1 |

Note: Analyses of BSL ratings on the item level revealed that the scores of the two HC participants reporting a moderate level of BPD symptom severity were driven primarily by items capturing unspecific psychological distress (e.g., "During the last week...I experienced myself as helpless"). Since increased unspecific stress levels were common in many healthy people due to the special burden of the corona pandemic and the lockdown at the time of the study, we did not exclude these participants from data analysis.

# Table S1b Number of participants with a severity of BPD features (VEI-BOR) below and above the cut-off for clinical relevance

| Severity category | Range of  VEI-BOR scores |  |  | In treatment | |
| --- | --- | --- | --- | --- | --- |
|  |  | HC | BPD | HC | BPD |
| Below cut-off | < 38 | 67 | 15 | 1 | 7 |
| Above cut-off | > 37 | 0 | 60 | 0 | 44 |

# F. Summary Statistics of 2×2×2 ANOVA

# Table S2 Results of the 2×2×2-ANOVA for ratings of intensity and confidence in social judgments

|  | ***Intensity*** | | | | ***Confidence*** | | | |
| --- | --- | --- | --- | --- | --- | --- | --- | --- |
|  | *F* | *p* |  | η_p_^2^ | *F* | *p* |  | ηp^2^ |
| Group | 22.38 | < .001 | *** | .14 | 6.53 | .012 | * | .05 |
| Task | 54.59 | < .001 | *** | .28 | 19.09 | < .001 | *** | .12 |
| Group × task | 13.00 | .001 | ** | .09 | 0.10 | .755 |  | < .01 |
| Mask | 201.30 | < .001 | *** | .59 | 155.90 | < .001 | *** | .53 |
| Group × mask | 1.67 | .198 |  | .01 | 4.18 | .043 | * | .03 |
| Mask × task | 44.71 | < .001 | *** | .24 | 14.46 | < .001 | *** | .09 |
| Group × task × mask | 1.21 | .274 |  | .01 | 4.63 | .033 | * | .03 |

*Note. df1* = 1, *df2* = 140.

# Summary Statistics of 2×2 ANOCVA in the BPD sample with BSL-23 covariate

# Table S3 Results of the 2×2-ANCOVA for ratings of intensity and confidence in social judgments in the BPD group with severity of BPD symptoms (BSL-23) as covariate

|  | ***Intensity*** | | | | ***Confidence*** | | | |
| --- | --- | --- | --- | --- | --- | --- | --- | --- |
|  | *F* | *p* |  | ηp^2^ | *F* | *p* |  | ηp^2^ |
| BSL | 11.91 | < .001 | *** | .14 | 0.14 | .705 |  | < .01 |
| Task | 6.15 | .015 | * | .08 | 9.73 | .003 | ** | .12 |
| Task × BSL | 1.01 | .317 |  | .01 | 0.16 | .690 |  | < .01 |
| Mask | 87.44 | < .001 | *** | .55 | 70.85 | < .001 | *** | .49 |
| Mask × BSL | 3.43 | .068 | (*) | .05 | 0.77 | .383 |  | .01 |
| Mask × task | 35.66 | < .001 | *** | .33 | 21.51 | < .001 | *** | .23 |
| Mask × task × BSL | 0.40 | .531 |  | <.01 | 0.82 | .367 |  | .01 |

*Note. df1* = 1, *df2* = 73; BSL: BSL-23 scores (*z*-transformed).

BPD symptom severity influenced the social judgments (main effect BSL): higher

BSL scores were associated with lower ratings of happiness and trustworthiness (*r* = -.12, *p* = .306). At trend level, BSL symptom severity was associated with the effects of MNCs on social judgments (interaction effect Mask × BSL): higher BSL scores were marginally significantly associated with larger differences between ratings of faces with and without MNCs (*r* = -.21, *p* = .068). To further explore the nature of this effect, we compared the correlation coefficients of the BSL scores with ratings of faces without and with MNC. This analysis revealed that a higher severity of BPD symptoms resulted at trend level in a stronger attenuation of intensity ratings for faces with MNC (*r* = -.41, *p* < .001) compared with faces compared with faces without MNC (r = -.24, *p* = .038, comparison between correlation coefficients: *z* = 1.63, *p* = .052). There was no effect of the severity of BPD symptoms on the participants’ confidence without MNC (*r* = -.24, *p* = .038, comparison between correlation coefficients: *z* = 1.63, *p* = .052). There was no effect of the severity of BPD symptoms on the participants’ confidence. Please see Figure S2 for illustration of the intensity ratings corresponding to the different levels of symptom severity.


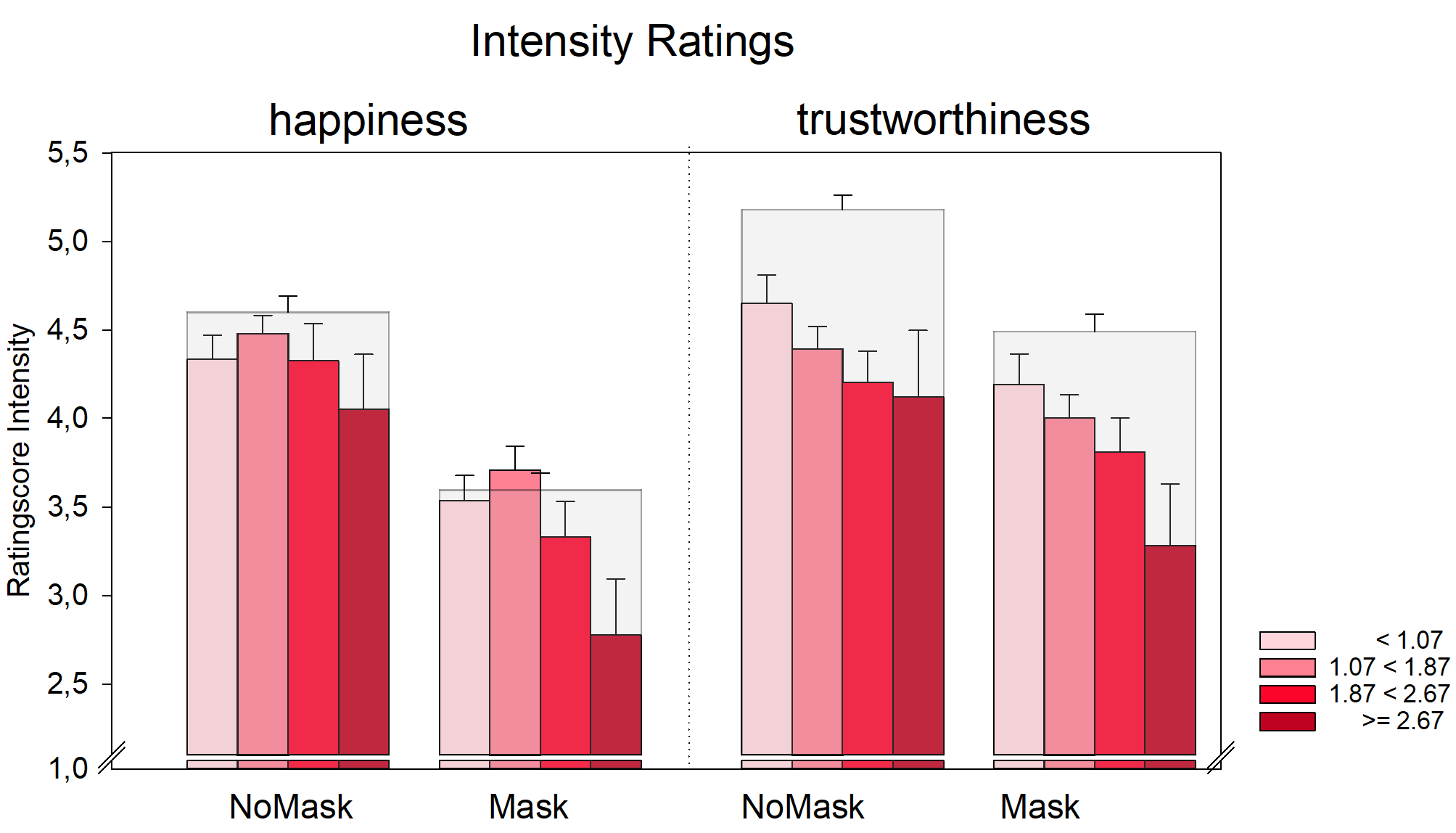


# Fig. S2 Ratings of happiness and trustworthiness for faces with and without MNCs depending on BSL-23 symptom severity categories in the BPD group. Categories correspond to 1) none to mild, 2) moderate, 3) high and 4) high to extremely high with grey bars corresponding to the ratings in the HC group.

# Relationships between Social Judgments and Different Facets of Wearing MNCs

# Table S4 Change in social judgements induced by MNCs and the evaluation of different facets of wearing MNCs

|  | HC | | BPD | |
| --- | --- | --- | --- | --- |
|  | *r_s_* | *p* | *r_s_* | *p* |
| Change happiness |  |  |  |  |
| Somatic burden | .19 | .134 | .14 | .235 |
| Emotional burden | .04 | .735 | .03 | .797 |
| Relief | .18 | .149 | -.07 | .566 |
| Protective benefit | -.04 | .734 | .08 | .504 |
| Compliance | -.06 | .623 | -.01 | .940 |
| Change trustworthiness |  |  |  |  |
| Somatic burden | .01 | .937 | .12 | .287 |
| Emotional burden | .18 | .156 | -.01 | .949 |
| Relief | -.05 | .683 | -.09 | .437 |
| Protective benefit | .03 | .821 | -.07 | .546 |
| Compliance | -.04 | .727 | .10 | .416 |

*Note.* The relationship is reported using Spearman correlations.

# Table S5 Trustworthiness ratings and the evaluation of different facets of wearing MNCs in the BPD group

|  | *Correlation* | | | *Partial correlation*  *(BSL-23 as covariate)* | | |
| --- | --- | --- | --- | --- | --- | --- |
|  | *r_s_* | *p* |  | *r_s_* | *p* |  |
| Somatic burden | -.40 | < .001 | *** | -.36 | .002 | ** |
| Emotional burden | .19 | .110 |  | .21 | .080 | (*) |
| Relief | .26 | .026 | * | .31 | .006 |  |
| Protective benefit | .29 | .010 | * | .31 | .008 | ** |
| Compliance | .32 | .005 | ** | .40 | <.001 | *** |

*Note.* Spearman correlations (*r_s_*) and comparison of correlation coefficients between groups are reported.

# Table S6 Happiness ratings and the evaluation of different facets of wearing MNCs

|  | HC | | | BPD | | |
| --- | --- | --- | --- | --- | --- | --- |
|  | *r_s_* | *p* |  | *r_s_* | *p* |  |
| Somatic burden | -.07 | .592 |  | -.07 | .580 |  |
| Emotional burden | -.15 | .215 |  | .05 | .670 |  |
| Relief | -.11 | .369 |  | .16 | .165 |  |
| Protective benefit | -.22 | .081 | (*) | -.02 | .882 |  |
| Compliance | -.04 | .774 |  | .11 | .333 |  |

*Note.* The relationship is reported using Spearman correlations.

# Table S7 Confidence in social judgments, its change induced by MNCs and the evaluation of different facets of wearing MNCs

|  | HC | | | BPD | | |
| --- | --- | --- | --- | --- | --- | --- |
|  | *r_s_* | *p* |  | *r_s_* | *p* |  |
| Change happiness |  |  |  |  |  |  |
| Somatic burden | -.15 | .224 |  | .14 | .233 |  |
| Emotional burden | .21 | .087 | (*) | .22 | .054 | (*) |
| Relief | -.17 | .179 |  | -.15 | .194 |  |
| Protective benefit | .04 | .778 |  | -.19 | .101 |  |
| Compliance | .04 | .742 |  | -.01 | .948 |  |
| Change trustworthiness |  |  |  |  |  |  |
| Somatic burden | -.14 | .269 |  | .04 | .758 |  |
| Emotional burden | .28 | .021 | * | .19 | .108 |  |
| Relief | -.21 | .087 | (*) | .07 | .539 |  |
| Protective benefit | -.08 | .534 |  | .03 | .795 |  |
| Compliance | -.11 | .390 |  | .09 | .430 |  |
| Happiness |  |  |  |  |  |  |
| Somatic burden | -.07 | .593 |  | -.17 | .156 |  |
| Emotional burden | -.06 | .614 |  | -.21 | .065 |  |
| Relief | .09 | .459 |  | .03 | .823 |  |
| Protective benefit | .07 | .562 |  | .18 | .113 |  |
| Compliance | -.03 | .832 |  | .11 | .334 |  |
| Trustworthiness |  |  |  |  |  |  |
| Somatic burden | .00 | .975 |  | -.11 | .369 |  |
| Emotional burden | -.06 | .645 |  | -.14 | .240 |  |
| Relief | .12 | .321 |  | -.02 | .843 |  |
| Protective benefit | .04 | .757 |  | .16 | .179 |  |
| Compliance | .09 | .468 |  | .14 | .223 |  |

*Note.* The relationship is reported using Spearman correlations. Please note that none of the correlations is significant at *p <.050* or as a trend (*p* < .100) after Benjamini-Hochberg correction.

# Effect of Childhood Abuse and Neglect on Social Judgments and Confidence During Judgements in BPD

Background and research question: The prevalence of adverse childhood experiences (ACE) in BPD is above 50% [11]. Studies that analyze the influence of ACE on trustworthiness appraisals and emotion recognition in BPD are missing. Several studies suggest that ACE such as sexual abuse, or physical and emotional abuse and neglect are linked to a negative bias when judging emotional expressions and to higher distrust [12-15]. In contrast, other studies suggest that individuals with ACE show an inaccurate identification of specific intimate partner betrayals, an inability to engage in proper self-protection [16, 17] or even reveal an extreme willingness to trust others, including untrustworthy persons, thus increasing the risk for further violations [18]. We investigated whether ACE are a factor modulating alterations of social judgments in BPD. We hypothesized that a higher severity of childhood traumatization exaggerates the severity of alteration in social judgements in BPD.

Methods: The influence of the severity of ACE on the effects of MNC and the type of social judgment in the BPD group was analyzed with a 2x2 ANCOVA (‘mask’, ‘task’) including the mean-centered CTQ scores as covariate.

Results The severity of child traumatization influenced the differences between social judgments (interaction effect CTQ × task, see Table S8). Comparing the correlation coefficients of the CTQ with happiness and trustworthiness ratings revealed that the level of traumatization resulted in a stronger attenuation of trustworthiness (*r* = -.21, *p* = .076) compared with happiness (*r* =.06, *p* = .640) ratings (*z* = -1.94, *p* = .027). There was no effect of the severity of child traumatization on the participants’ confidence (see Table S8).

# Table S8 Results of the 2×2-ANCOVA for ratings of intensity and confidence in social judgments with CTQ as covariate

|  | ***Intensity*** | | | | ***Confidence*** | | | |
| --- | --- | --- | --- | --- | --- | --- | --- | --- |
|  | *F* | *p* |  | ηp^2^ | *F* | *p* |  | ηp^2^ |
| CTQ | 0.76 | .388 |  | .01 | 0.45 | .507 |  | < .01 |
| Task | 6.41 | .013 | * | .08 | 9.73 | .003 | ** | .12 |
| CTQ × task | 4.16 | .045 | * | .05 | 0.11 | .745 |  | < .01 |
| Mask | 85.48 | < .001 | *** | .54 | 70.35 | < .001 | *** | .49 |
| Mask × CTQ | 1.71 | .195 |  | .02 | 0.25 | .621 |  | < .01 |
| Task × mask | 35.52 | < .001 | *** | .33 | 21.37 | < .001 | *** | .23 |
| Task × mask × CTQ | 0.11 | .743 |  | < .01 | 0.34 | .561 |  | < .01 |

*Note. df1* = 1, *df2* = 73; CTQ = *z*-transformed scores of the Childhood Trauma Questionnaire.

Conclusions: A history of more severe adverse childhood experiences in BPD affected social judgments independently of hiding social cues in the lower part of the face by an MNC. Our findings revealed that trustworthiness ratings were reduced more strongly in those individuals of the BPD group who reported a higher level of ACE compared with happiness ratings supporting previous findings [12-15].

# References

1. Walker EA, Unutzer J, Rutter C, Gelfand A, Saunders K, VonKorff M, et al. Costs of health care use by women HMO members with a history of childhood abuse and neglect. Arch Gen Psychiatry. 1999;56:609-13.

2. Bohus M, Kleindienst N, Limberger MF, Stieglitz R-D, Domsalla M, Chapman AL, et al. The short version of the Borderline Symptom List (BSL-23): development and initial data on psychometric properties. Psychopathology. 2009;42:32-9.

3. Groves JA, Engel RR. The German Adaptation and Standardization of the Personality Assessment Inventory (PAI). J Pers Assess. 2007;88:49-56.

4. Morey LC. The Personality Assessment Inventory professional manual. Odessa: Psychological Assessment Resources; 1991.

5. Hautzinger M, Keller F, Kühner C. BDI-II Beck Depressions-Inventar. Frankfurt a. M.: Harcourt Test Services; 2006.

6. Beck AT, Steer RA, Brown GK. Manual for the Beck Depression Inventory-II. San Antonio: Psychological Corporation; 1996.

7. Klinitzke G, Romppel M, Hauser W, Brahler E, Glaesmer H. Die deutsche Version des Childhood Trauma Questionnaire (CTQ) - psychometrische Eigenschaften in einer bevölkerungsrepräsentativen Stichprobe [The German Version of the Childhood Trauma Questionnaire (CTQ): psychometric characteristics in a representative sample of the general population]. Psychother Psychosom Med Psychol. 2012;62:47-51.

8. Bernstein DP, Stein JA, Newcomb MD, Walker E, Pogge D, Ahluvalia T, et al. Development and validation of a brief screening version of the Childhood Trauma Questionnaire. Child Abuse Negl. 2003;27:169-90.

9. Beierlein C, Kemper CJ, Kovaleva A, Rammstedt B. Kurzskala zur Messung des zwischenmenschlichen Vertrauens: Die Kurzskala Interpersonales Vertrauen (KUSIV3). GESIS - Working Papers. 2012;22:1-26.

10. Watson D, Wiese D, Vaidya J, Tellegen A. The Two General Activation Systems of Affect: Structural Findings, Evolutionary Considerations, and Psychobiological Evidence. Journal of Personality and Social Psychology. 1999;76:820-38.

11. Porter C, Palmier‐Claus J, Branitsky A, Mansell W, Warwick H, Varese F. Childhood adversity and borderline personality disorder: a meta‐analysis. Acta Psychiatr Scand. 2020;141:6-20.

12. Ardizzi M, Martini F, Umiltà MA, Evangelista V, Ravera R, Gallese V. Impact of childhood maltreatment on the recognition of facial expressions of emotions. PLoS One. 2015;10:e0141732.

13. Bernath MS. The effects of child maltreatment and parental empathy on children's trust: A multidimensional analysis [dissertation]. Los Angeles: University of California; 1997.

14. Catalana A, Díaz A, Angosto V, Zamalloa I, Martínez N, Guede D, et al. Can childhood trauma influence facial emotion recognition independently from a diagnosis of severe mental disorder? Rev Psiquiatr Salud Ment (Engl Ed). 2020;13:140-9.

15. Hepp J, Schmitz SE, Urbild J, Zauner K, Niedtfeld I. Childhood maltreatment is associated with distrust and negatively biased emotion processing. Borderline Personal Disord Emot Dysregul. 2021;8:5.

16. Gobin RL, Freyd JJ. Betrayal and revictimization: Preliminary findings. Psychol Trauma: Theory Res Pract Policy. 2009;1:242-57.

17. Gobin RL, Freyd JJ. The impact of betrayal trauma on the tendency to trust. Psychol Trauma: Theory Res Pract Policy. 2014;6:505-11.

18. Zurbriggen EL, Freyd JJ. The link between child sexual abuse and risky sexual behavior: The role of dissociative tendencies, information-processing effects, and consensual sex decision mechanisms. In: Koenig LJ, Doll LS, O'Leary A, Pequegnat W, editors. Child sexual abuse to adult sexual risk: Trauma, revictimization, and intervention. Washington, DC: American Psychological Association; 2004. p. 135-57.

1. ^1^Development of the Interdisciplinary Affective Science Laboratory (IASLab) Face Set was supported by the

   National Institutes of Health Director’s Pioneer Award (DP1OD003312) to Lisa Feldman Barrett. More information is available online at www.affective-science.org. [↑](#footnote-ref-1)
